# Non-invasive real-time biopsy of intracranial lesions using short time expanded circulating tumor cells on glass slide: report of two cases

**DOI:** 10.1186/s12883-016-0652-x

**Published:** 2016-08-08

**Authors:** N. Malara, G. Guzzi, C. Mignogna, V. Trunzo, C. Camastra, A. Della Torre, A. Di Vito, A. M. Lavecchia, M. Gliozzi, C. Ceccotti, G. Volpentesta, A. Lavano, G. Donato, V Mollace

**Affiliations:** 1 IRC-FSH Laboratories Department of Health Science, University Magna Graecia, 88100 Catanzaro, Italy; 2 Bionem Laboratories, Department of Experimental and Clinical Medicine, University “Magna Graecia”, 88100 Catanzaro, Italy; 3 Neurosurgery Unit, Department of Medical and Surgery Science, University “Magna Graecia”, 88100 Catanzaro, Italy; 4 Pathological Anatomy, Department of Health Science, University “Magna Graecia”, 88100 Catanzaro, Italy; 5 Cellular Toxicological Laboratory. Department of Health Science, Salvatore Venuta Campus, University “Magna Graecia”, 88100 Catanzaro, Italy; 6 Department of Experimental and Clinical Medicine, University “Magna Graecia”, 88100 Catanzaro, Italy; 7 Anatomical and Pathological Sciences, Pugliese-Ciaccio Hospital, 88100 Catanzaro, Italy; 8 Neurosurgery Unit, Pugliese-Ciaccio Hospital, 88100 Catanzaro, Italy

**Keywords:** Circulating tumor cells, Short-term expansion, Intracranial tumor

## Abstract

**Background:**

Circulating Tumor Cells (CTCs) are promising biomarkers for monitoring solid cancer and were used to monitor brain tumors. Here we report two cases in which, for the first time, CTCs were used in cytological diagnostic evaluation to discriminate a space-occupying lesion of the brain.

**Case presentation:**

Two cases of focal intracranial lesions, unclassified for diagnosis, untreated and apparently symptomatic, were examined after high-contrast resolution Magnetic Resonance Imaging and/or Computed Tomography scans. CTCs were seeded on chamber slides and short-time expanded under the optimized conditions as we previously reported. The first case was a focal lesion localized in the parietal-occipital area in a 67-year-old woman. The second case was a 31-year-old man with an expansive intracerebral lesion localized in the left peri-trigonal area. Both patients underwent excisional biopsy. Histopathological evaluation of the biopsy confirmed the previous cytological diagnoses, and the analysis of the clinical outcomes retrospectively validated both diagnoses.

**Conclusions:**

The cases here reported illustrate the potential for using expanded CTCs as non-invasive, real-time biopsy. Moreover, non-invasive real-time biopsy can represent an alternative diagnostic tool to be used when a functional area of the brain is at risk of injury from excisional biopsy procedures.

## Background

The use of new technologies to detect Circulating Tumor Cells (CTCs) in the bloodstream of cancer patients has resulted in a better understanding of the biology of solid cancer and related circulating progeny [1]. It now appears clear that the primary tumor generates circulating progeny not only during the phase of disseminated malignancy [2]. Many studies have shown that this phenomenon occurs in each phase of cancer development [3]. Isolated tumor cells have been detected in the bone marrow of up to 16 % of the American Joint Committee on Cancer stage I and II of breast cancer [4, 5]. CTCs come from the initial phase of a tumor and from eventual metastasis. CTCs are able to go back to the primary tumor site and therefore participate in the formation of relapsing lesions [6]. Up to now, different hypotheses have tried to explain their presence in the peripheral blood of patients affected by intracranial malignancies [7]. In fact, the intracerebral localization implicates the interaction of the tumor bulk and its cancer cell progeny with the Blood Brain Barrier (BBB). The BBB consists in an endothelial wall, where cells are connected by tight junctions, surrounded by astrocytic end-feet with pericytes embedded in the vessel basal membrane. Moreover, neurons and microglia are involved in the cytoarchitecture of BBB. Therefore, the first condition for the cancer cell progeny to intravasate seems to be closely associated with altered BBB permeability. In theory, focal disturbances in the BBB could happen in every stage of intracranial malignancies development, due to inflammatory conditions or direct interaction with tumor cells [8, 9]. Study on glioblastoma cancer showed that glioma cells displace, or even eliminate, astrocytic end-feet and directly enter in contact with endothelial cells. These interactions result in a breach of BBB that justify the CTCs detection. CTCs have been described only in cases of advanced glioblastoma [10] and, in literature, the actual delay between the initial tumor diagnosis and metastasis ranges between 1 and 60 months [11].

In intracranial pathologies, a further limit in the use of CTCs as biomarkers is set by the prevalent detection of CD326 expression through the most used technique, Cell Search. Indeed, tumor cells in endocranial tumors lose or do not express CD326 [12]. As a result, research has been carried out by analyzing peripheral blood in patients affected by endocranial tumors and seeking circulating markers such as tumoral DNA or regulatory mRNA or oncoproteins [13]. Recent research has shown that CTCs isolated from patients with glioblastoma are enriched with mesenchymal markers [14].

We have previously described an optimized protocol to isolate and short-time expand CTCs derived from cancer patients [1, 15].

**Fig. 1:**
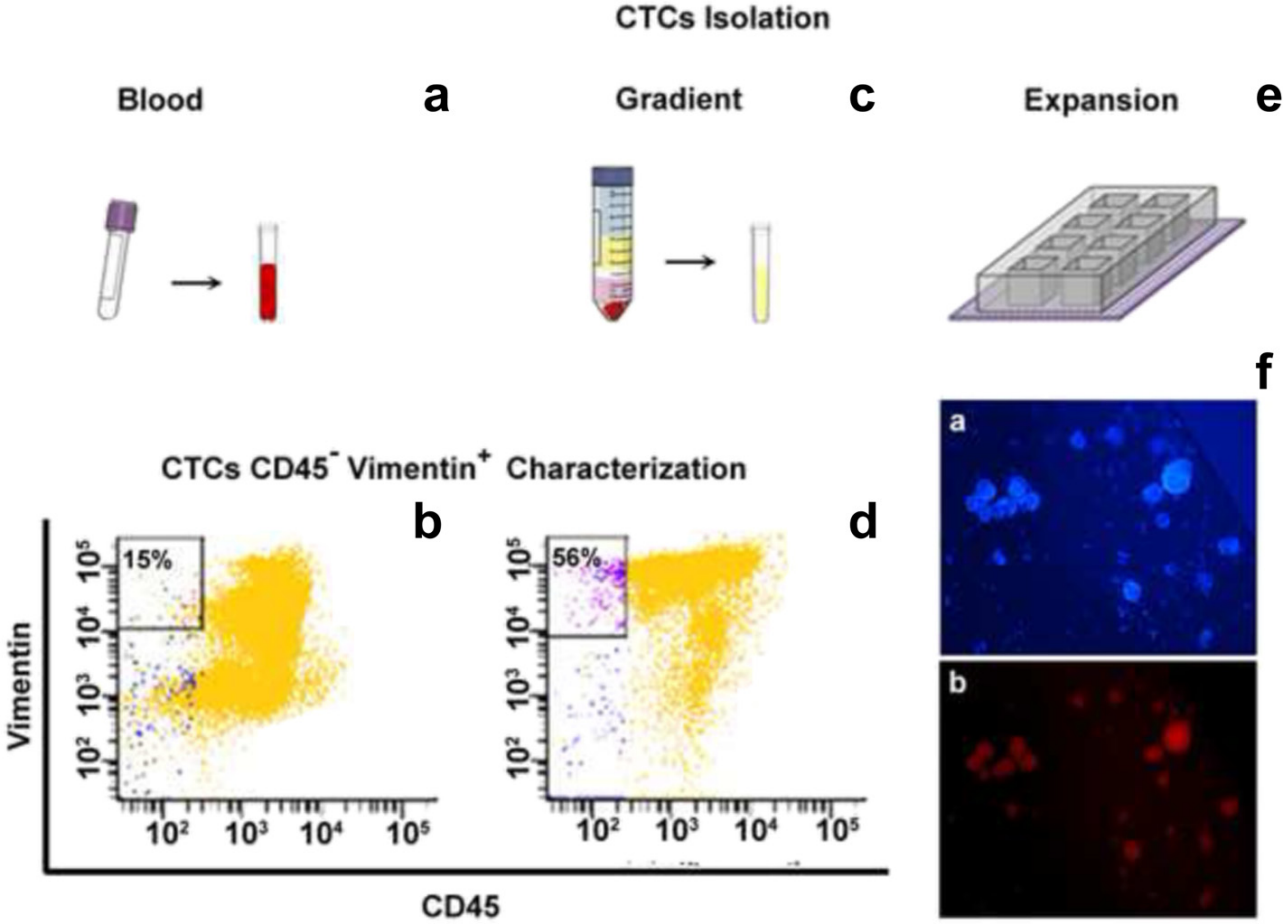
CTCs isolation procedure. CTCs isolation was performed on a whole blood sample previously treated with a solution for lysing red blood cells (**a**), and on a specific phase (after gradient procedure performed centrifuging the peripheral blood mounted on Ficoll) (**c**). Peripheral blood sample is achieved by searching for cells expressing Vimentin. The reported dot plots in **b**) (obtained from the analysis of whole blood sample) vs **d**) (obtained from the analysis after gradient procedure) confirm that the specific phase is enriched for CTCs. Finally, the CTCs were seeded on slide chamber and expanded for 14 days (**e**). Cultivated CTCs were stained for immunofluorescence analysis (**f**) highlighting the nuclei in blue, DAPI stained (a), and in red Vimentin expression (b). Scale bar: 200 μm

The protocol involves the isolation of CTCs from peripheral blood as shown in Fig. 1(a-f). As described in previous papers, we have identified a specific density phase for CTCs in peripheral blood [15, 16]. In intracranial cases reported here, we verified that the previous identified phase was enriched with CTCs (Fig. 1a-d). The cells isolated from this phase are then put to seeding, as previously described [1, 15, 16], in a specific culture medium and expanded for 14 days (Fig. 1e). The short-time cultivation protocol has been optimized in the course of many experiments which have allowed us to pinpoint the time required to show up the proliferative capacity of the transformed cells and to preserve their original phenotypes. Our approach does not make any selection of phenotypes during the initial phase, in order to save the heterogeneous composition of the CTCs. In fact, the circulating tumor progeny we expect to be made up of cancer cells with an epithelial or mesenchymal or hybrid epithelial-mesenchymal phenotypes. Our purpose here is only to highlight the proliferation capability, which is typical of a transformed cell when compared to a normal cell. The chamber-slides are not pre-treated in order to promote the cells with high survival and proliferative capacities. The slides with cells in adhesion are useful for pathological analysis. Furthermore, the cells, which have grown in adhesion and suspension, can be classified according to the mesenchymal antigen vimentin expression (Fig. 1f).

Here, we report two cases of intracranial tumor whose sizes range between 0,5–4 cm, and in these cases we have isolated CTCs and recovered them on chamber-slides for a short-time culture. CTC-slides were then used for cytological evaluation [17]. These cases represent the first example of how CTCs can be used as biomarkers in the diagnostic phase of tumors such as the endocranial ones, for which the current imaging techniques do not guarantee a definitive diagnosis and the patient undergoes delicate biopsy procedures.

## Case presentation

### Case 1

**Fig. 2:**
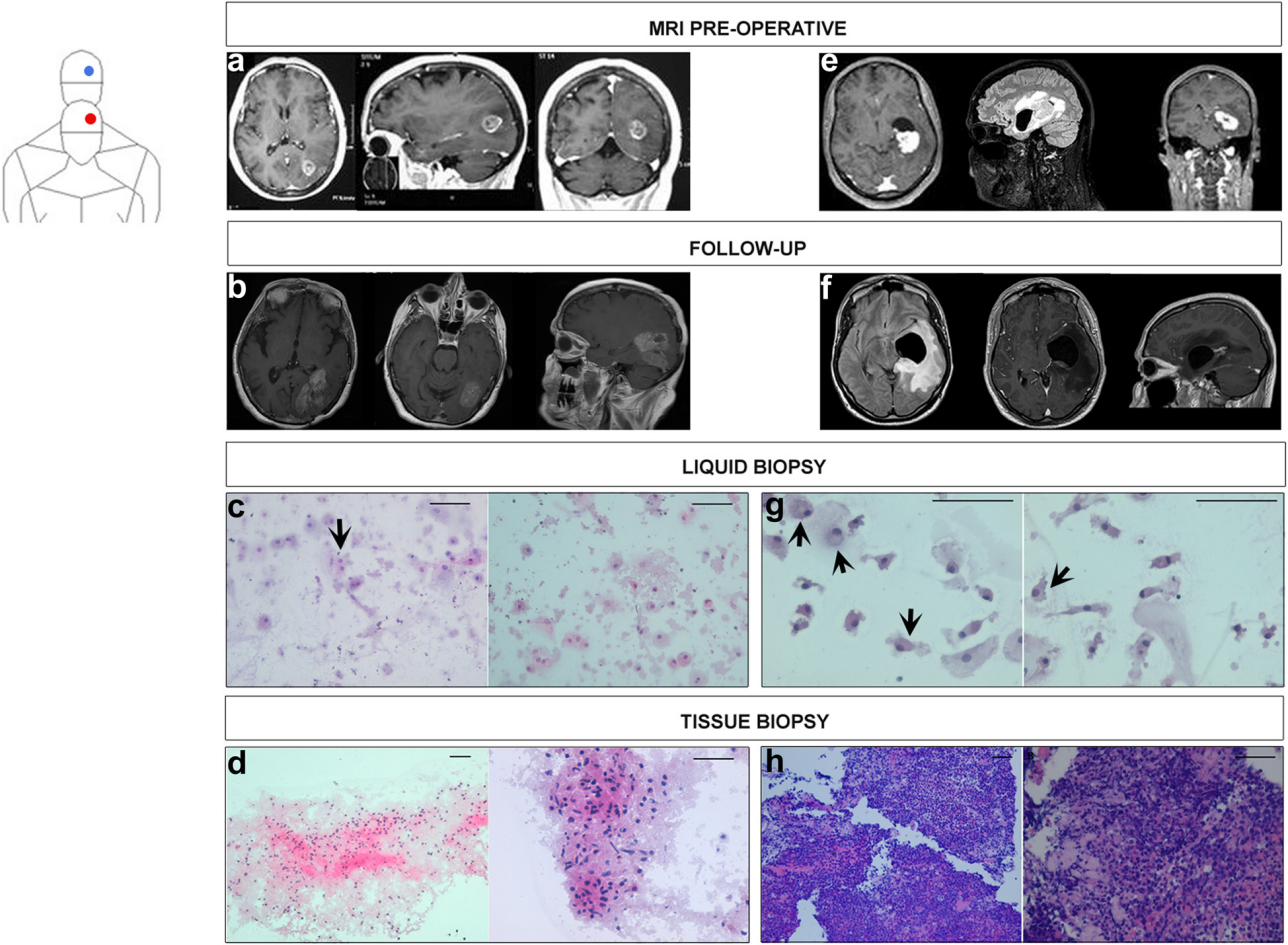
Clinical cases. Case 1 (sequence **a**-**d**) is shown in red and case 2 is shown in blue (sequence **e**-**h**). The MRIs were carried out during the clinical course of preliminary diagnosis (**a** and **e**) with liquid biopsy (**c** and **g**). Diagnosis was subsequently confirmed by conventional tissue biopsy (**d** and **h**). Finally, MRIs as part of the follow-up were respectively reported for the two cases (**b** and **f**). Case 1. In **a** pre-operative MRI of the head shows an expansive lesion with peritrigonal localization. The lesion dislocates the ventricular system with relative dilatation of the left temporal horn. The parenchymal side of the lesion, after contrast infusion, shows a strong enhancement in respect to the perilesional oedema. Para-sagittal and coronal sections after contrast perfusion show the lesion proximal to the IV cerebral ventricle. In **b** MRI, performed during the follow up, shows relapsing lesion. In **c** Liquid biopsy, H&E staining of short-time cultivated CTCs shows elements with gemistocytic pattern and moderate nuclear atypia. The black arrow shows a cluster of cells with eosinophilic cytoplasm. Scale bars: 100 μm. In **d** Tissue biopsy, H&E staining of histologic glioblastoma specimen. Please note the necrosis, the atypical astrocytic cell proliferation, and the gemistocytic features. Scale bars: 100 μm. Case 2. In **e** pre-operative MRI of the head shows acystic expansion entrapping the temporal horn of the left lateral ventricle with controlateral dislocation of the ventricular system. In **f** MRI, performed during the follow up after treatment shows left-sided cyst and dilated ventricle with reduction of contrast-enhanced MRI of the primary lesion. In **g** H&E staining of cytological elements on slides obtained by short-time expansion of CTCs. Please note large, round elements with abundant cytoplasm, indicated by four black arrows. Scale bars: 100 μm. In **h** H&E staining of excisional biopsy shows Diffuse Large B-Cell Lymphoma. Scale bars: 100 μm

In November 2013 a 67-year-old female with negative familiar history for any type of tumor lamented asthenia, mental confusion, memory disturbances, dizziness, decreased vision and partial right homonymous hemianopsia. These symptoms gradually worsened over a period of a few days. The patient was referred to us for observation accusing the above symptoms and she underwent a complete neurological investigation. The results of the investigation excluded a deficiency in the cranial nerves and an Magnetic Resonance Imaging (MRI) scan was requested. A pre-operative MRI was performed which highlighted an expansive lesion altering the signals at the left temporal lobe and the left posterior occipital lobe (Fig. 2a). The presence of oedema determined compression of the occipital horn of the lateral ventricle. Moreover, extensive alteration of the signal from the left temporal, occipital and posterior parietal lobes, with swollen appearance of the parenchyma and resulting compression on the occipital horn were reported. The lesion was mainly located in the left occipital region showing a circular, ring-like distribution of the contrast.

A ring contrast-enhanced lesion localized in the left occipital region with intralesional hypointensity are not pathognomonic for glioblastoma. At the same time the site, the compression on the ventricle, the oedema and the persistent symptoms made the patient a candidate for surgical intervention to remove the lesion tissues. The initial patient’s performance status according to the Eastern Cooperative Oncology Group (ECOG) was classified as ECOG = 1. During the pre-operative phase, as well as the routine blood tests, a sample of peripheral blood in EDTA was taken for analysis of the CTCs. Peripheral blood sample is collected to detect CTCs. We used whole peripheral blood pre-treated with a red cells lysis solution (Fig. 1a) and in parallel we performed gradient procedure (Fig. 1c) as previously reported [15]. The reported dot plots in Fig. 1b) vs d), confirm that the gradient phase is enriched in CTCs Vimentin positive (Vimentin^pos^) as previously demonstrated for other type of tumors [1, 15, 16].

After 14 days of CTCs expansion, the chambers were eliminated and the adherent cancer cells were fixed with Cytofix aerosol preparation for pathological analysis. The pathologist diagnosed an high grade astrocytic tumor due to neoplastic cells with gemistocytic pattern and nuclear atypia, in Haematoxylin and Eosin (H&E) stained slide (Fig. 2c).

In December 2013 the patient underwent a bulk resection of the lesion: a left parietal-occipital craniotomy was performed and no complications were found after surgery. Post-surgery recovery was satisfying, the management of the patient during the post-surgical phase did not present significant problems; antioedema and antibiotic therapies were administered and the patient recovery time was 10 days. The patient was dismissed 10 days after surgery. The clinical symptoms disappeared except for the persistent right homonymous hemianopsia. The cancer tissue excised during surgery was analysed by a pathologist (Fig. 2d).

The pathologist diagnosed a multiform glioblastoma due to neoplastic tissue with severe atypical astrocyte proliferation in presence of mitosis and microvascular proliferation. This diagnosis was confirmed using immunohistochemistry techniques.

The specialists advised adjuvant “whole brain” radiotherapy. Total radiotherapy dose was 60 Gy delivered in 30 consecutive days associated with temozolomide.

The patient tolerated the antitumor therapy well without showing modification of the initial performance.

In January 2014 a second peripheral blood sampling for CTCs isolation was performed. The cytological analysis of the resulting slide highlighted the persistence of CTCs in the peripheral blood, with an increased concentration of 7 CTCs-Vimentin^pos^/ml, compared to the first blood sampling (4,5 CTCs-Vimentin^pos^/ml).

After 9 month of apparent wellness and clinical benefit, in October 2014 the patient returned to us complaining difficulty in maintaining a standing position, urinary incontinence and further reduction in vision that led to complete hemianopsia. A brain MRI revealed a recurrence of the tumor which showed as an irregular area with increased absorption of the contrast. In particular, T2-hyperintense and T1-hypointense highlighted perifocal oedema in left parietal-temporal-occipital area. The ipsilateral occipital horn appeared partially dislocated, while the hippocampal cortex, the hippocampus and the midbrain appeared dislocated to the left, with a slight shift of the midline (Fig. 2b). The patient was treated with anti-oedema agents, mannitol and cortisone to reduce the symptoms. Only in February 2015 the patient agreed to undergo surgery to excise the relapsing lesion. The surgeons carried out a second surgical resection of the left parietal-occipital lesion. Conditions of the patient in the post-surgical phase were complicated by the formation of a fistula containing liquid on the operation site. Anti-oedema and antibiotic therapies were administered. The histopathologic examination was diagnostic for a recurrence of a glioblastoma. The patient’s general state of health began to worsen and she died in March 2015.

### Case 2

In May 2015 a 31-year-old man presented with a one-month history of headaches associated with phonophobia and photophobia, dizziness and short term memory deficits. On admission, he was somnolent and disoriented. He had bilateral papilledema and left homonymous hemianopsia.

Laboratory examinations showed 14500/mm^3^ white blood cell count, 558 UI/Lactate dehydrogenase (LDH). Cerebrospinal fluid analysis showed high values in proteins and glucose whereas the cytology was negative. Computed Tomography (CT) scans showed a cystic expansion entrapping the temporal horn of the left lateral ventricle with an extensive periventricular hypodensity area and controlateral dislocation of the ventricular system.

Preoperative imaging demonstrated that the primitive lesion displayed a high density on CT scans. Contrast-enhanced MRI showed a T1-hypointense and T2-hyperintense lesion. The signals were significantly enhanced by the contrast (Fig. 2e).

The patient was advised to undergo stereotactic biopsy procedures. During the pre-operative phase, a blood sample was collected for CTCs detection. The resulting slides were analysed by the pathologist who suspected a lymphoma for the morphology of neoplastic cells highlighted by H&E staining (Fig. 2g). The patient underwent CT scans with and without contrast for the upper abdomen, the lower abdomen and the thorax. Scans confirmed that the lymphoma was exclusively localized in the intracranial area. In this case, the CTCs were not characterized for mesenchymal antigens as reported for the first case. Given the cytological analysis and neuroimaging data, diffuse large B-cell lymphoma diagnosis was directly confirmed by immunostaining for CD20 on tissue biopsy specimen. Therapy with high doses of corticosteroids resulted in a nearly complete disappearance of the primary lesion and improvement in the patient’s general state of health. We made a stereotactic biopsy of the primary temporal lesion and stereotactic placing of external ventricular shunt by a ventricular catheter positioned into the left temporal horn. The patient responded well in the post-operative phase and recovered within 3 days of surgery whilst continuing the anti-oedema therapy. During the second post-operative day there was an appreciable improvement in clinical symptoms. The neurological deficits completely disappeared after the therapy. Follow-up CT scans showed adequate decompression of the temporal horn and disappearance of the ventricular low-density area. The midline shift disappeared as well.

In the following days the patient underwent a whole body fluorodeoxyglucose (FDG)-positron emission tomography (PET) that, despite its spatial and biological limits of resolution, showed no areas of pathological increase in glucose metabolism in the body regions examined. FDG-PET brain did not recognize areas of significant alteration in glucose metabolism in various cortical, subcortical and subtentorial districts. These data confirm that the lymphoma is primitive and exclusively of the brain.

On histopathological examination of the surgical specimen (Fig. 2h), the initial hypothesis of a brain lymphoma was confirmed, revealing a CD20^pos^ large B cell lymphoma with high proliferation index (Ki67 Positivity) greater than 70 %. Subsequently, the patient was referred to our medical oncology outpatient clinic where he was followed carefully. The external ventricular shunt was removed because of the high risk of infection. Chemotherapy cycle was prescribed with the following scheme: rituximab 375 mg/mq on day 1, methotrexate 3500 mg/mq on day 2, cytosine arabinoside 2 gr/mq doses twice daily on day 2,3, thiotepa 30 mg/mq on day 4 (weight 78 kg, height 178 cm, body surface area 1,9 mq, total dose rituximab 700 mg, methotrexate 6650 mg, cytosine arabinoside15,2 g, thiotepa 60 mg). Treatment was generally well tolerated by the patient and currently (follow up after 8 months MRI in Fig. 2f) the patient’s general condition are good.

## Conclusions

In our limited experience, there are no obvious pathological difference between excisional and liquid biopsy in diagnostic evaluation of space-occupying brain lesions, as confirmed in the cases presented here. In the excisional biopsy, the tissue organization can address the diagnostic procedure better than the cytological samples obtained through the liquid biopsy. On the other hand, as demonstrated in these cases, this condition is not strictly needed to formulate a correct diagnosis. In the first case, we were able to identify the mesenchymal phenotype of circulating tumor cells, consisting in Vimentin expression. This information implemented with neuroimaging has strongly suggested the early diagnosis of high grade astrocytic tumor. This multidisciplinary approach, in both cases, was able to highlight the nature of the primary lesion. Moreover, in case 1, the Vimentin-positivity of CTCs was also used in a personalized monitoring of the patient, allowing an early detection of the disease recurrence.

Furthermore, many neurological disorders can mimic clinically a tumor and there are diseases in which the neuroimaging is not sufficient to solve diagnostic problems.

The cases reported here show the possibility of discriminating intracranial pathology through a simple blood test. The possibility of performing non-invasive biopsy procedures to obtain real time precious information can be considered an improvement for an early diagnosis of brain lesions.

In every phase of neoplastic pathology it is useful to have a source of tumor cells which permits the characterization of the same. In fact, during chemotherapy or radiotherapy, we apply selective pressure that modifies the tumor cells identified prior to administrating the antitumor therapies. The availability of CTCs allows us to perform a personalized monitoring of the disease progress via both tracking the number of cells and cell characterization. Moreover, the cost-effectiveness analysis of the management of these cases suggest that using CTCs as non-invasive real–time biopsy, the costs currently employed in analogue cases by Health Service can break down of 70 %.

The protocol proposed here guarantees, respect to other current methods, that the cell isolated are neoplastic and representative of the primary lesion. In this study, we focalize on the CTCs phenotype but, this protocol can be also used for genotyping, DNA/sRNA analysis, and external vesicle production, with the advantage to discriminate the cancer cells from contaminating hematological cells. More cases must be studied to confirm these preliminary results.

## Abbreviations

BBB, blood brain barrier; CT, computed tomography; CTC, circulating tumor cells; ECOG, Eastern Cooperative Oncology Group; FDG-PET, fluorodeoxyglucose-positron emission tomography; H&E, haematoxylin and eosin; LDH, lactate dehydrogenase; MRI, magnetic resonance imaging; Positive, pos
